# Supplementary material for: A Comprehensive Assessment of Ultraviolet-Radiation-Induced Mutations in Flammulina filiformis Using Whole-Genome Resequencing
Source: J Fungi (Basel). 2024 Mar 20;10(3):228. doi: 10.3390/jof10030228 (PMC10971301; doi:10.3390/jof10030228)
Supplement: Supplementary file 1 [file jof-10-00228-s001.zip › Supplementary Material S8/KEGG annotation/out/64381550635650.os/KO/out_map/map04650.html]

KEGG PATHWAY: Natural killer cell mediated cytotoxicity - Reference pathway


|  |  |
| --- | --- |
| **Natural killer cell mediated cytotoxicity - Reference pathway** |  |

[
Pathway menu
| Organism menu
| Pathway entry
| Show description
| User data mapping
]

Natural killer (NK) cells are lymphocytes of the innate immune system that are involved in early defenses against both allogeneic (nonself) cells and autologous cells undergoing various forms of stress, such as infection with viruses, bacteria, or parasites or malignant transformation. Although NK cells do not express classical antigen receptors of the immunoglobulin gene family, such as the antibodies produced by B cells or the T cell receptor expressed by T cells, they are equipped with various receptors whose engagement allows them to discriminate between target and nontarget cells. Activating receptors bind ligands on the target cell surface and trigger NK cell activation and target cell lysis. However Inhibitory receptors recognize MHC class I molecules (HLA) and inhibit killing by NK cells by overruling the actions of the activating receptors. This inhibitory signal is lost when the target cells do not express MHC class I and perhaps also in cells infected with virus, which might inhibit MHC class I exprssion or alter its conformation. The mechanism of NK cell killing is the same as that used by the cytotoxic T cells generated in an adaptive immune response; cytotoxic granules are released onto the surface of the bound target cell, and the effector proteins they contain penetrate the cell membrane and induce programmed cell death.

|  |  |
| --- | --- |
| Reference pathway | 100% |
